# A traditional gynecological medicine inhibits ovarian cancer progression and eliminates cancer stem cells via the LRPPRC–OXPHOS axis

**DOI:** 10.1186/s12967-023-04349-3

**Published:** 2023-07-26

**Authors:** Ruibin Jiang, Zhongjian Chen, Maowei Ni, Xia Li, Hangjie Ying, Jianguo Fen, Danying Wan, Chanjuan Peng, Wei Zhou, Linhui Gu

**Affiliations:** 1 Zhejiang Cancer Hospital, Hangzhou Institute of Medicine (HIM), Chinese Academy of Sciences, Hangzhou, 310022 Zhejiang China; 2 Hangzhou Institute of Medicine (HIM), Chinese Academy of Sciences, Zhejiang 310022 Hangzhou, People’s Republic of China

**Keywords:** Ovarian cancer, Oxidative phosphorylation, Gossypol acetic acid, LRPPRC

## Abstract

**Background:**

Ovarian cancer (OC) is the most lethal malignant gynecological tumor type for which limited therapeutic targets and drugs are available. Enhanced mitochondrial oxidative phosphorylation (OXPHOS), which enables cell growth, migration, and cancer stem cell maintenance, is a critical driver of disease progression and a potential intervention target of OC. However, the current OXPHOS intervention strategy mainly suppresses the activity of the electron transport chain directly and cannot effectively distinguish normal tissues from cancer tissues, resulting in serious side effects and limited efficacy.

**Methods:**

We screened natural product libraries to investigate potential anti-OC drugs that target OXPHOS. Additionally, LC-MS, qRT-PCR, western-blot, clonogenic assay, Immunohistochemistry, wound scratch assay, and xenograft model was applied to evaluate the anti-tumor mechanism of small molecules obtained by screening in OC.

**Results:**

Gossypol acetic acid (GAA), a widely used gynecological medicine, was screened out from the drug library with the function of suppressing OXPHOS and OC progression by targeting the leucine-rich pentatricopeptide repeat containing (LRPPRC) protein. Mechanically, LRPPRC promotes the synthesis of OXPHOS subunits by binding to RNAs encoded by mitochondrial DNA. GAA binds to LRPPRC directly and induces LRPPRC rapid degradation in a ubiquitin-independent manner. LRPPRC was overexpressed in OC, which is highly correlated with the poor outcomes of OC and could promote the malignant phenotype of OC cells in vitro and in vivo. GAA management inhibits cell growth, clonal formation, and cancer stem cell maintenance in vitro, and suppresses subcutaneous graft tumor growth in vivo.

**Conclusions:**

Our study identified a therapeutic target and provided a corresponding inhibitor for OXPHOS-based OC therapy. GAA inhibits OC progression by suppressing OXPHOS complex synthesis via targeting LRPPRC protein, supporting its potential utility as a natural therapeutic agent for ovarian cancer.

**Supplementary Information:**

The online version contains supplementary material available at 10.1186/s12967-023-04349-3.

## Introduction

Ovarian cancer (OC) is a leading cause of cancer-related mortality in women, contributing to more than 204 thousand diagnosed cases worldwide annually. Despite significant advances in chemotherapy, surgery, and radiotherapy procedures, the fatality rates of patients with OC remain high, with a 5-year survival rate of < 30% [1–3]. Identifying novel markers of disease progression and effective therapeutic drugs remains an urgent clinical requirement to improve treatment outcomes.

Metabolic reprogramming is a hallmark of cancers, and identifying drugs targeting tumor metabolism is a research hotspot [4–7]. The deficiency of mitochondrial oxidative phosphorylation (OXPHOS) and coordinated upregulation of glycolysis (also known as the Warburg effect) has long been recognized as a fundamental feature of tumor metabolism [8]. Recent studies have challenged this view and reestablished the essential role of OXPHOS in cancer. Cancer cells are reported to harbor robust mitochondrial function during tumor progression and contain increased mtDNA content compared with their normal tissue counterparts, including ovarian cancer [9, 10], lung cancer [11, 12], and so on. Moreover, cancer cells resistant to chemotherapy [13] or certain targeted therapies [14] are more inclined to OXPHOS, concomitant with elevated mitochondrial mass and oxygen consumption. Numerous studies have further shown that cancer stem cells, which are responsible for metastasis and recurrence, prefer OXPHOS to glycolysis [15]. Alterations in OXPHOS have been reported in OC, with a significant impact on patient survival and maintenance of cancer stem cells [3]. Highly invasive ovarian cancer cells preferentially use glutamine instead of glucose to supplement the TCA cycle, which markedly improves the oxygen consumption rate [10]. These collective findings suggested that targeting of OXPHOS is a promising therapeutic approach to delay ovarian cancer progression.

Attempts in OXPHOS-targeted therapy so far have mainly focused on either directly suppressing the activity of OXPHOS complexes or inhibiting key enzymes in the tricarboxylic acid cycle (TCA, the metabolic pathway providing reducing equivalents and electrons for OXPHOS), such as metformin [16], buformin [17], and IACS-010759 [18]. However, as non-transformed cells also depend on OXPHOS, such inhibitors also affect the metabolism of normal cells, leading to toxic and side effects. Identification of drugs that specifically inhibit OXPHOS is of significant clinical value for the effective management of ovarian cancer.

In this study, we performed a high-throughput screening of 850 natural compounds to identify small inhibitory molecules of OXPHOS in OC cells. The traditional gynecologic drug, gossypol acetic acid (GAA), was identified as an inhibitor of cellular OXPHOS and tumor cell growth in vitro and in vivo. Mechanistically, GAA suppressed OXPHOS by targeting leucine-rich pentatricopeptide repeat-containing protein (LRPPRC). Immunohistochemical and reanalysis of the transcriptomic in the TCGA database revealed overexpression of LRPPRC in OC. Overexpressed LRPPRC negatively correlated with the overall survival of OC, representing a new OC tumor marker. Upregulation of LRPPRC elevated the synthesis of electron transport chain complex subunits encoded by mtDNA and promoted OXPHOS. Knockdown of LRPPRC inhibited the proliferation of OC cells and led to a depletion of tumor stem cells in vitro and reduced subcutaneous tumorigenesis in vivo. GAA interacted directly with LRPPRC and induced its degradation in a proteasome-independent manner, resulting in OXPHOS deficiency. GAA treatment suppressed malignant phenotypes of OC cells significantly, both in vitro and in vivo. Most importantly, GAA inhibited the synthesis of new OXPHOS complexes but not the function of existing OXPHOS complexes. These characteristics provide an OXPHOS therapeutic strategy targeting cancer cells that overexpressed LRPPRC and proliferate rapidly, with minimal damage to slowly proliferating normal cells. Therefore, we proposed that OXPHOS inhibition by targeting LRPPRC by GAA was a promising treatment strategy for OC.

## Materials and methods

### Cell cultures

OC cell lines, A2780 and SKOV3, were purchased from the American Type Culture Collection (ATCC). A2780 cells were cultured in RPMI1640 with 10% fetal bovine serum (Gibico, Grand Island, USA). SKOV3 cells were cultured in McCoy’s 5 A medium with 10% fetal bovine serum (Gibico). Cells were screened using the Cell Culture Contamination Detection Kit (Thermo Fisher Scientific, Massachusetts, USA) and shown to be negative for mycoplasma contamination. All cells were grown in a humidified incubator at 37 ℃ under 5% CO_2_.

### Patients and tissue samples

OC tissues were obtained from Zhejiang Cancer Hospital (Hangzhou, China). All samples were collected with informed consent and stored in a biobank at − 80℃ until analysis. Written informed consent was obtained from each patient, and ethical approval was acquired from the Ethics Committee of Zhejiang Cancer Hospital. Survival analyses were conducted with the online tool KM Plotter ( https://kmplot.com/analysis/ ). Patients with ovarian cancer (n = 107) were selected for the overall survival assay, and the Log-rank value was automatically computed. The LRPPRC level in OC based on tumor grade was analyzed using UALCAN ( http://ualcan.path.uab.edu/index.html ). The level of LRPPRC in OC based on IHC was analyzed using the Human protein atlas ( https://www.proteinatlas.org/ ).

### Natural compounds screen

Natural product libraries were purchased from Topscience (Shanghai, China), and the compounds were listed in Additional file 2. Cells were added to wells in 96 well plates, and then add natural compounds into wells; the final concentration of the drug is 10 µM. Cell viability was detected by CCK8 after 48 h, and the most lethal drugs (TOP 30) were used for subsequent screening. Mitochondrial membrane potential was measured using Mito-Tracker Red CMXRos (Beyotime Biotechnology, Shanghai, China). Briefly, PDC cells were treated with different compounds for 48 h, incubated with Mito-Tracker Red (100 nM) for 30 min, and then washed with PBS. Then, 4% paraformaldehyde solution was used to fix the cells. The cells were observed under a fluorescence microscope and the fluorescence intensity was measured by Image J.

### Immunohistochemistry

For immunohistochemistry (IHC) analysis, tumor sections were deparaffinized by Xylene and rehydrated with solutions with gradient concentrations of ethanol aqueous. Endogenous peroxidase activity was blocked with 3% hydrogen peroxide. Heat-induced antigen retrieval was performed in a citrate buffer (0.01 M, pH 6.0, 95 ℃) using a steamer. Slides were incubated with primary antibodies against LRPPRC (1:250 dilution), CDK4 (1:200), CDK6 (1:200), or Ki-67 (1:10,000) overnight at 4 ℃ in a moist chamber. After washing thoroughly, sections were incubated with Dako EnVision + System HRP-Labeled Polymer for 30 min at room temperature, counterstained with hematoxylin, dehydrated, coverslipped, and visualized.

### qRT-PCR analysis

Total RNA was extracted with TRIzol reagent (Invitrogen, California, USA) according to the operation manual. cDNA was generated using a commercial kit (Takara, Dalian, China); the reaction program was listed below: 37 ℃ for 15 min, 85 ℃ for 15 s, and stored at 4 ℃. Real-time PCR was performed with an SYBR kit (Takara) using an ABI7500 Fast Real-Time PCR system (Applied Biosystems, California, USA). The primer sequences are listed in Additional file 3.

### Proteomic analysis and metabolomics analysis

A2780 cells were treated with GAA for 48 h, and then cells were harvested. Filter-aided sample preparation method was applied for protein digestion [19]. LC/MS/MS analysis was conducted in a nano-LC & Q-Exactive system as we reported previously [20]. All the raw files were searched against the UniProt human protein sequence database in Maxquant (version 1.6). Finally, a *t*-test was performed to see which proteins were significantly changed between the groups, and an adjusted *p*-value (also named *q*-value) was calculated. Proteins in the data frame > 1.5 changes and the *q*-value < 0.01 were marked as significantly changed. Glycolysis and TCA cycle metabolite analyses were performed in Suzhou PANOMIX Biomedical Tech Co., LTD. For details, see Additional file 4.

### Bioenergetic measurements

Cellular bioenergetic measurements were performed using the Seahorse XFe96 Analyzer (Agilent Technologies, California, USA). For drug treatment studies, cells were seeded at a density of 20,000 cells/well and exposed to GAA for 24 h. On the day of the assay, the cell culture medium was removed and replaced with a medium supplemented with 10 mM glucose, 1 mM sodium pyruvate, and 2 mM L-glutamine for the XF Cell Mito Stress Test. The XF Mito Stress Test assay medium was supplemented with 1 mM sodium pyruvate and 2 mM L-glutamine. The XF Cell Mito Stress Test was employed to determine mitochondrial flux when the basal oxygen consumption of cells was measured, followed by sequential injection of the ATP synthase inhibitor oligomycin (5 µM; port A), the uncoupler fluoro-carbonyl cyanide phenylhydrazone (FCCP, 2 µM; port B), and the complex I inhibitor rotenone (0.5 µM; port C) combined with the complex III inhibitor antimycin A (0.5 µM; port D). For evaluation of glycolytic flux using the XF Glycolysis Stress Test, non-glycolytic acidification was determined, followed by sequential injection of glucose (10 mM; port A) to assess basal glycolysis, the ATP synthase inhibitor oligomycin (5 µM; port B), and the hexokinase inhibitor 2-deoxy-glucose (50 mM; port C).

### Western blot analysis

Cells were collected via centrifugation at 800 *g* for 5 min and lysed in RIPA lysis buffer (Beyotime, Jiangsu, China) containing 1 mM phenylmethylsulfonylfluoride. 30 µg lysates from each sample were loaded onto 10% SDS polyacrylamide gels for immunoblot analysis. The primary antibodies used were listed below: anti-LRPPRC (Santa Cruz, USA, 1:1000), anti-LC3A/B (CST, USA, 1:1000), anti-Lonp1 (Proteintech, China, 1:1000), anti-SLIRP (Proteintech, China, 1:1000) anti-GAPDH (CST, USA, 1:2000), anti-α-Tubulin (CST, USA, 1:1000), total OXPHOS WB antibody cocktail (Abcam, USA, 1:1000), land anti-β-actin (CST, USA, 1:1000).

### Clonogenic assay

1000–5000 cells were seeded in 6-well plates and treated with different concentrations of GAA for 48 h. Cells were washed twice with a complete medium and further cultured in an incubator at 37 ℃ for 10–14 days. Colonies > 50 cells were considered viable and scored using an inverted microscope after staining with crystal violet.

### Wound scratch assay

Cultured cells in the confluent monolayer were wounded using a needle to scratch the surface, followed by exposure to GAA. Cell movement and initial wounds were imaged at 0 h, 24 h, and 48 h after GAA administration and then analyzed using Image J software.

### Tumor cell spheroidization

The method according to the previous reports [21]. Briefly, cells were resuspended (5000 cells/mL) and added to low adhesion 24 well plates with specific medium. The medium was DMEM/F12 (Gibico, Grand Island, USA) supplemented with 2% B-27, 20 ng/ml EGF (SinoBiological, Beijing, China) and 20 ng/ml bFGF (SinoBiological, Beijing, China). After a week of cultivation, the cell spheroidization were captured and measured by microscope. Sphere Formation Efficiency (SFE) was calculated according to the following formulas:

SFE  = Sphere count **÷** Total cells.

### Cell cycle analysis

Cells (2.0 × 10^5^) were seeded in six-well plates and allowed to adhere for 24 h and then treated with various concentrations of GAA for 48 h. Cells were harvested by trypsin digestion and fixed with 70% ethanol at − 4 ℃ overnight. After thoroughly washing with PBS, cells were incubated with the propidium iodide solution (100 µg/mL RNase A, 50 µg/mL propidium iodide) at 37 °C for 30 min. Cell cycle analysis was conducted via flow cytometry with CXP software, and the results were evaluated with Cytomics™ FC 500 software (Beckman).

### In vivo experiment

BALB/c nude mice (age 4 weeks, body weight, 16–18 g) were purchased from SLAC (Shanghai, China). All experiments were performed according to the guidelines of the National Institutes of Health Guide for Care and Use of Laboratory Animals. To evaluate the function of LRPPRC, A2780 cells engineered with shRNA silencing of LRPPRC (2 × 10^6^ cells in 100 µL of 1:1/PBS Matrigel) were injected into flanks of mice. Three weeks later, mice were sacrificed and tumors were isolated. To evaluate the anti-tumor effect of GAA, A2780 cells were injected into flanks, and GAA was administrated when tumor volume reached about 100 mm^3^. Animals were randomly divided into control (normal saline) and GAA (30 mg/kg, every other day). Three weeks later, mice were sacrificed and tumors were isolated for further analysis.

### Statistical analysis

Statistical significance was examined using GraphPad Prism software. All data are presented as means ± SD. Statistical differences between the two groups were determined using the Student’s *t*-test with one-way analysis of variance (ANOVA). *P* values < 0.05 were considered statistically significant.

## Results

### Screening compounds that suppress OXPHOS and cell viability in ovarian cancer

**Fig. 1:**
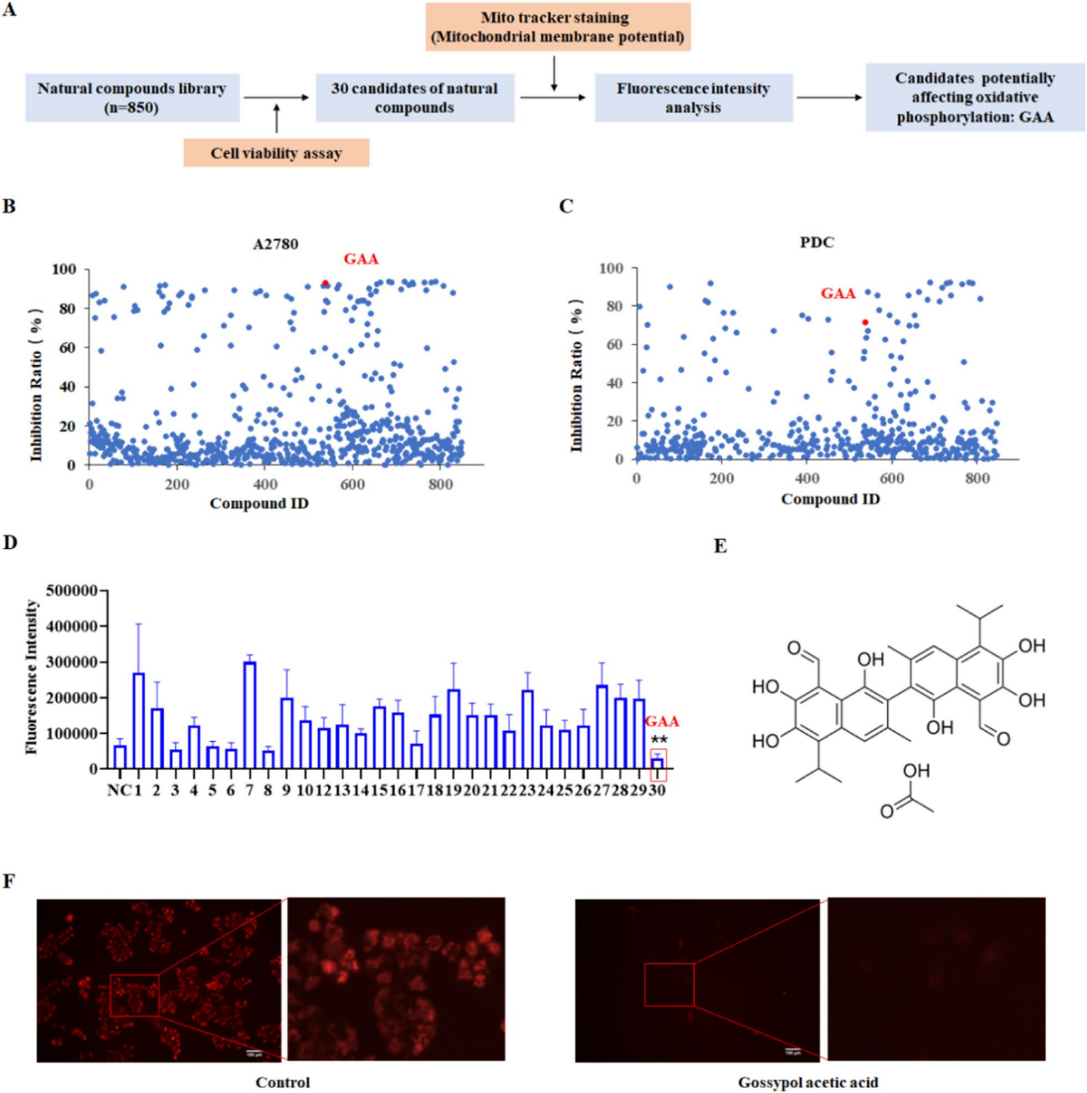
GAA affects mitochondrial function in ovarian cancer cells. **A** High-throughput screening of OXPHOS inhibitors based on natural compounds in ovarian cancer cells. GAA was screened as a candidate OXPHOS inhibitor. **B**, **C** Cell inhibition rates determined using MTT following treatment with natural compounds (n=852). **D** Relative fluorescence intensity in A2780 cells treated with candidate natural compounds. GAA vs NC, n=3, **P<0.01. **E** Chemical constituents of gossypol acetic acid (GAA). **F** Measurement of mitochondrial membrane potential via Mito Tracker staining in A2780 cells treated with GAA

OXPHOS is reported to play an essential role in ovarian cancer, particularly in the metabolism of tumor cells with drug resistance. We designed a two-step screening system to identify potential small molecules that could suppress OXPHOS and cell viability (Fig. 1A). In the first step screen, A2780 and patient-derived cell (PDC) were used for screening. Cells were co-cultured with drug candidates and subjected CCK8 assay to measure each small-molecule drug’s effect on cell activity. The compounds that could inhibit the activity of ovarian cancer were screened again for their inhibition effect on OXPHOS activity by MitoTracker Red staining assay. Mito Tracker dye can detect mitochondrial membrane potential and indirectly reflect mitochondrial oxidative phosphate. After the two-step screening, we could obtain compounds that may inhibit ovarian cancer cell activity by targeting the OXPHOS process. A total of 850 natural compounds with biological activity were included in our system (Fig. 1B, C). In the first step screening, 30 of the 850 compounds exhibited a cell viability inhibition rate of at least 70% at a concentration of 10 micromoles, including Dioscin, Gracillin Crocin II, and so on (Additional file 1: Table S1). In the second step of screening, 30 compounds were treated with PDC for 48 h, and Mito Tacker was used to detect Mitochondrial membrane potential (Additional file 5: Fig. S1). Only the gossypol acetate (GAA) treatment showed a significantly decreased MitoTracker signal in OC cells (Fig. 1D–F). GAA is a gynecologic drug used for uterine diseases, but its biological function has not been studied in ovarian cancer. After the systematic screening, we selected GAA for further investigation.

### GAA suppresses the malignant phenotypes of OC cells in vitro and in vivo

**Fig. 2:**
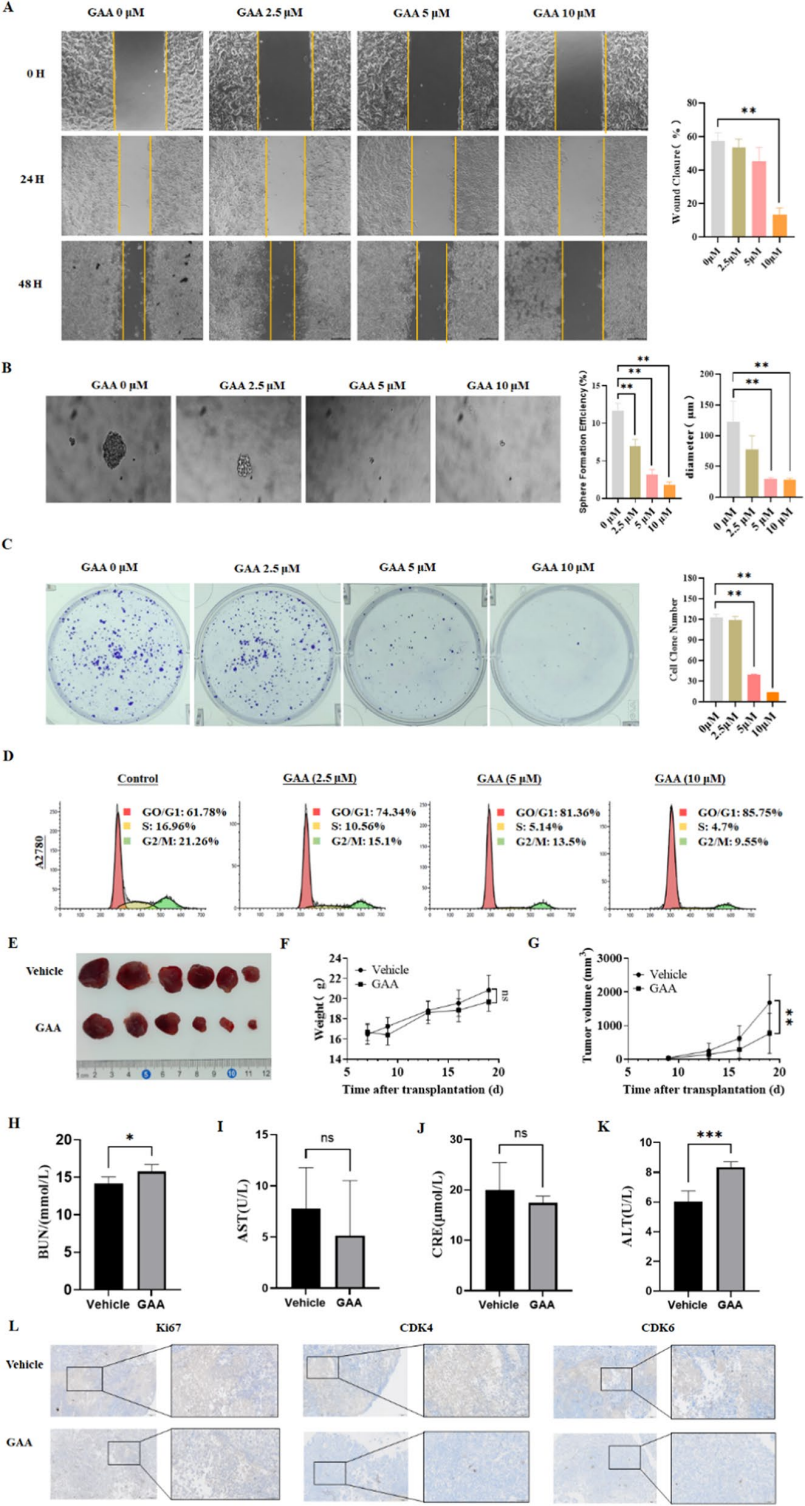
GAA suppresses the malignant phenotypes of OC cells in vitro and in vivo. **A** Wound healing assay of A2780 cells treated with GAA for 0 h, 24 and 48 h. **B** Cell spheroidization assay of A2780 cells treated with GAA. **C** Clonogenic assays of A2780 cells treated with GAA for 48 h. **D** Flow cytometry analysis showing cell cycle changes in A2780 cells treated with GAA for 48 h. **E**–**G** Image showing tumor inhibition by GAA, with measurements of tumor weight and volume. **H**–**K** Graphs showing changes in serum levels of BUN, AST, CRE, and ALT in the mouse model. **L** IHC results of the staining patterns of Ki-67, CDK4, and CDK6 proteins in tumors

We then tested the anti-tumor effect of GAA using OC cells in vitro. In the cell scratch assay, GAA reduced cell confluence at the scratch site in a dose- and time-dependent manner. 2.5 µM GAA was enough to reduce the degree of confluence by nearly 50% in SKOV3 cells (Additional file 5: Fig. S2A), indicating GAA could suppress the migration ability of OC cells (Fig. 2A, Additional file 5: Fig. S2A). The suspension culture system was used to identify and enrich tumor stem cells. The administration of GAA could significantly decrease the number of naked-eye visible tumor spheres in the suspension culture system, indicating GAA could effectively consume ovarian cancer tumor stem cells (Fig. 2B, Additional file 5: Fig. S2B). The colony formation assay also showed that the number of cell clones was also significantly decreased after GAA addition (Fig. 2C, Additional file 5: Fig. S2C). In flow cytometry analysis, treatment with GAA significantly increased the proportion of OC cells in the G0/G1 phase, with minimal effect in inducing cell apoptosis (Fig. 2D, Additional file 5: Figs. S2D and S3A, B). Therefore, the suppressed colony formation caused by GAA might be attributed to the affected cell cycle and stemness, but not apoptosis.

To establish whether the therapeutic effects of GAA could be replicated in vivo, A2780 cells were injected into the flanks of BALB/c athymic nude mice. When tumor widths reached 3–5 mm, mice were regrouped and treated with GAA. Notably, GAA exerted a significant inhibitory effect on tumor growth in vivo (Fig. 2E). An apparent tumor volume reduction was observed after GAA treatment (Fig. 2G), and GAA-treated mice displayed no weight loss or liver or kidney function impairment, indicating the good safety of GAA in vivo (Fig. 2F, H–K). Additionally, in IHC experiments, GAA suppressed the expression of protein markers associated with tumor progression, including Ki67, CDK4, and CDK6 (Fig. 2L). Taken together, the small molecule GAA we screened out showed good anti-tumor activity against ovarian cancer both in vitro and in vivo.

### GAA suppresses the OXPHOS metabolism in OC cells

**Fig. 3:**
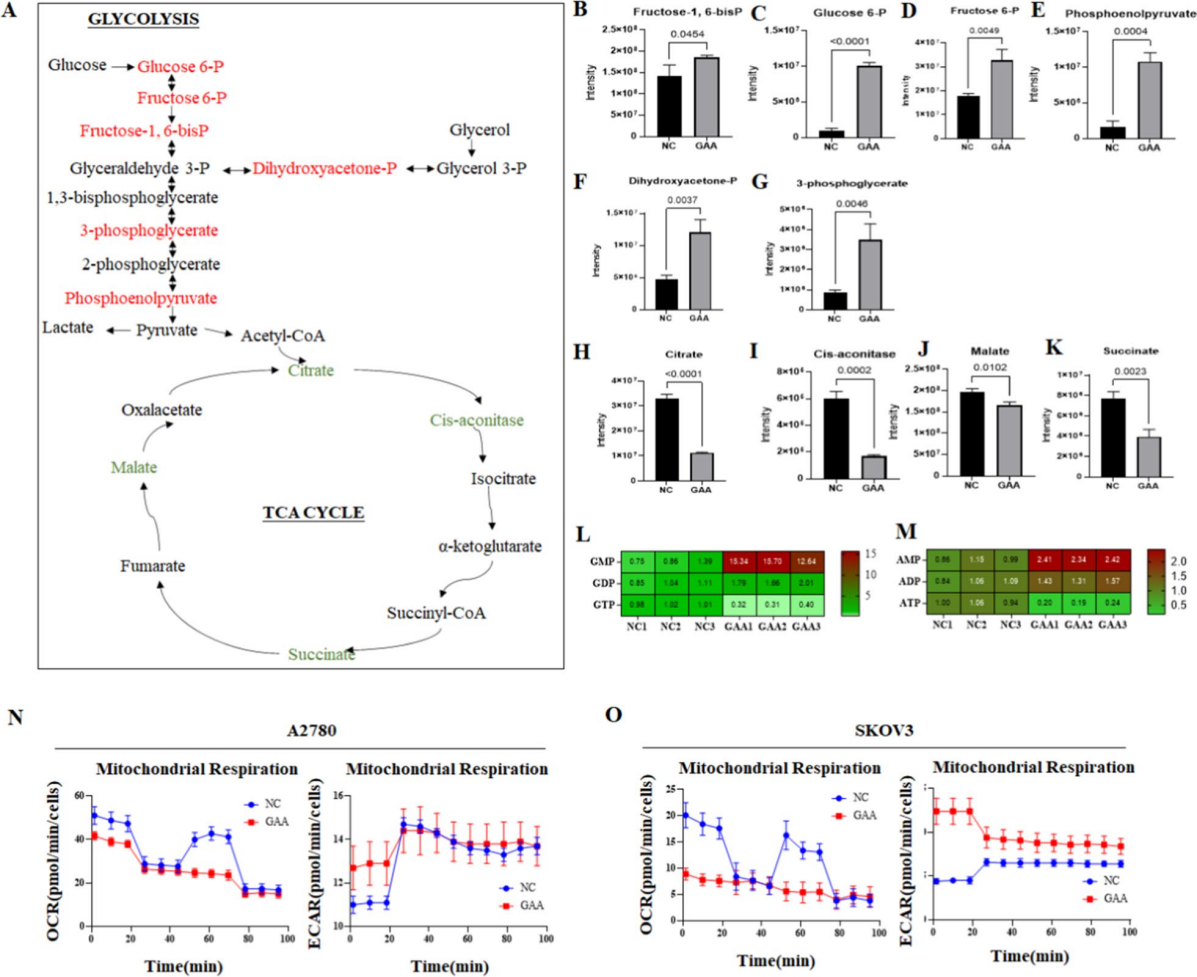
GAA suppresses the OXPHOS metabolism in OC cells. **A**–**K** Metabolites in glycolytic and TCA cycles detected in A2780 cells treated with GAA. **L**, **M** GTP and ATP was detected in A2780 cells treated with GAA. **N**, **O** OCR and ECAR measurements in A2780 and SKOV3 ovarian cancer cells treated with GAA (10 μM)

We then tested if the screened GAA could inhibit the OXPHOS in OC cells. Metabolomics was applied to detect changes in the abundance of intermediate products of carbon metabolism in the OC A2780 cells (Fig. 3A). Mass spectrometric analysis showed that intermediates belonging to the OXPHOS and TCA cycle were broadly downgraded after GAA treatment, including Citrate, Cis-aconitase, Succinate, and Malate (Fig. 3H–K). In contrast, the steady-state levels of most intermediates in the glycolysis pathway increased significantly in GAA-treated A2780 cells, including Glucose 6-P, Fructose 6-P, Fructose-1,6-bisP, Dihydroxyacetone-P, 3-phosphoglycerate and Phosphoenolpyruvate (Fig. 3B–G). Furthermore, the total amount of ATP and GTP in cells was also decreased by about 50% (Fig. 3L, M), indicating the occurrence of a cell energy crisis after GAA treatment.

To further check GAA-induced changes in mitochondrial oxygen consumption and ATP production, the oxygen consumption rate (OCR, an indicator of oxygen consumption caused by OXPHOS) was measured. In the OCR assay, three inhibitors against the OXPHOS process, oligomycin, FCCP, and antimycin A rotenone, were introduced to the cells in order. In the control cells (DMSO-treated), the addition of oligomycin inhibited the function of OXPHOS complex V, suppressing its electron transfer, thus lowering the OCR value. Adding an uncoupling agent, FCCP (Carbonyl cyanide 4-(trifluoromethoxy)phenylhydrazone), destroyed the mitochondrial membrane potential. Therefore, the electron transportation and OCR values reached the maximum after FCCP addition. At last, as antimycin A and rotenone inhibited the function of OXPHOS complex I, they completely shut down the OXPHOS process and caused a sharp decline in OCR value. In our experiment, treatment of OC cells (A2780 and SKOV3) with 5 µM GAA for 24 h resulted in a dramatic reduction in mitochondrial activity, with a significantly decreased basal OCR, ATP production, and Maximal OCR (Fig. 3N, O). Taken together, these findings suggest that GAA reduces the oxidative respiration capacity of mitochondria.

### GAA suppresses OXPHOS in OC by targeting LRPPRC

**Fig. 4:**
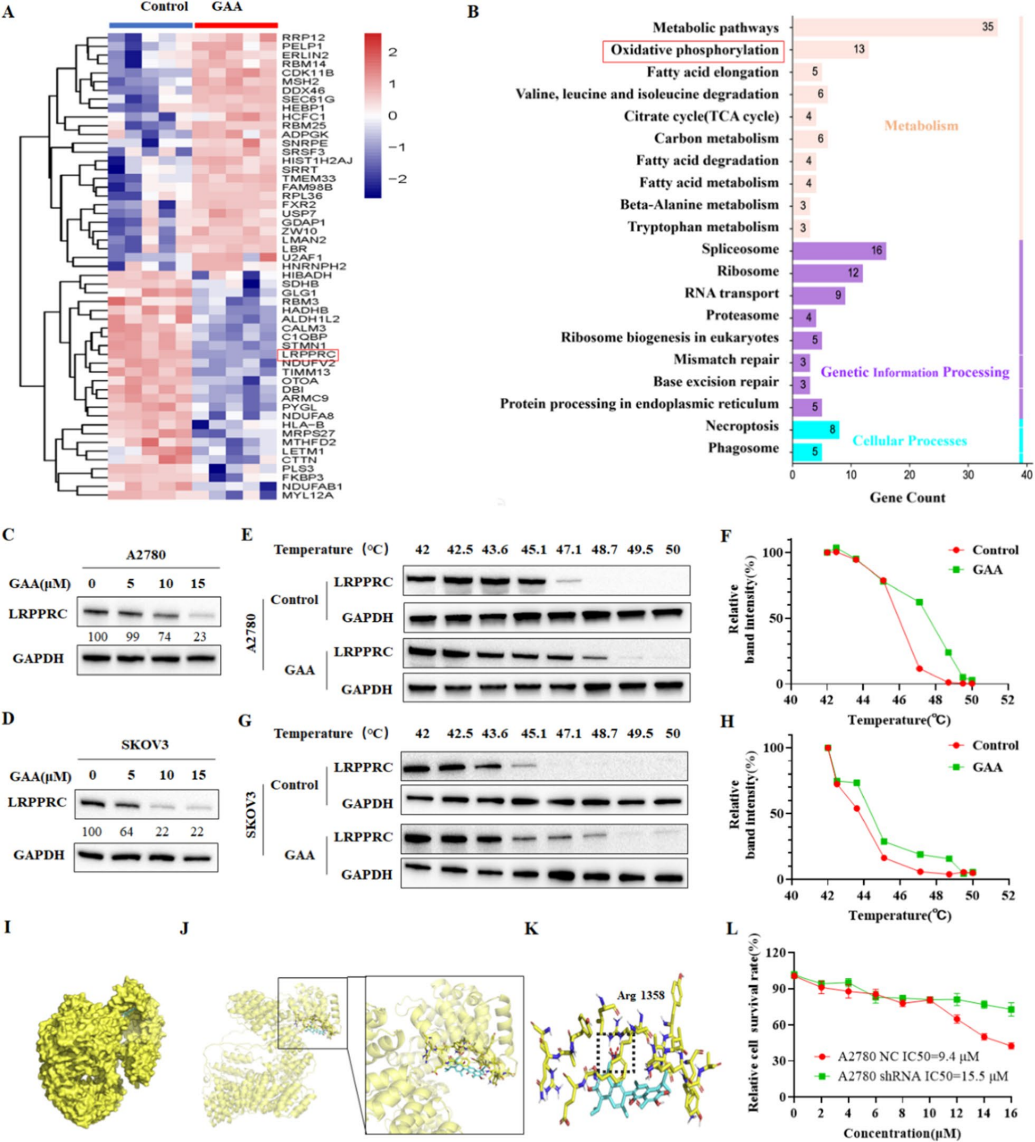
GAA suppresses OXPHOS in OC by targeting LRPPRC. **A** Heatmap showing changes in protein expression after GAA treatment. **B** KEGG analysis of genes significantly altered by GAA compared to control A2780 cells. **C**, **D** Western blot of LRPPRC protein levels in ovarian cancer cells following GAA treatment. **E**–**H** Examination of GAA-LRPPRC binding in A2780 and SKOV3 cells using cellular thermal shift and western blot assays. **I**–**K** Predicted binding mode of GAA to LRPPRC via docking analysis. **L** CCK8 detection of cell survival rates in GAA-treated A2780 cells with LRPPRC knockdowns

We then moved to torture the protein target of GAA, which responsible for the anti-tumor effect we observed in OC cells. We then applied proteomic analysis to identify proteins that were affected by GAA. A total of 54 proteins showed significant differential expression between control and GAA-treated groups (fold change ≥1.5 or ≤ 0.5, false discovery rate (FDR *q* value) ≤0.01, among which 26 were downregulated, and 28 were upregulated after GAA treatment (Fig. 4A). Enrichment analysis of differentially expressed genes was performed to reveal the signal pathways most significantly affected by GAA, and the oxidative phosphorylation pathway ranked second (Fig. 4B). The proteomic results also confirmed the significant effect of GAA on oxidative phosphorylation, consistent with our previous experimental results.

Among all the differentially altered proteins, we selected LRPPRC as a potential GAA protein target for further study for a list of reasons. First, mitochondrial OXPHOS complexes contain more than 100 proteins encoded by the genome in the nucleus and 13 proteins encoded by mitochondrial genes (mtDNA). LRPPRC binds to the mRNA of all 13 (mtDNA) encoded genes, enabling its stability and protein expression. Proteomics results demonstrated that the amount of LRPPRC protein was significantly reduced after GAA treatment. The decrease of LRPPRC expression in ovarian cancer cells was further confirmed by immunoblotting, in which GAA caused a concentration-dependent decrease of LRPPRC protein in both A2780 and SKOV3 cell lines. (Fig. 4C, D). Furthermore, the cellular thermal shift assays demonstrated a robust direct interaction between LRPPRC and GAA in A2780 and SKOV3. More specifically, western blot analysis showed the presence of LRRPRC at the lower test temperature, followed by its disappearance with increasing temperature in the control group. However, we observed a strong band of LRPPRC at 48.7 ℃, which was still detectable upon increasing the temperature to 50 ℃ after treatment of cells with GAA (Fig. 4E–H). Our molecular docking results indicate that GAA targets LRPPRC by forming hydrogen bonds with the amino group of Arg1358 (Fig. 4I–K). These results proved that GAA could bind to LRPPRC protein directly and decrease LRPPRC expression.

We also assess the molecular mechanisms responsible for the GAA-induced LRPPRC down expression. PCR results showed no significant decline in LRPPRC mRNA, suggesting post-transcriptional regulation of LRPPRC protein by GAA (Additional file 5: Fig. S5A, B). The cycloheximide (CHX)-based protein half-life time assay showed the half-time of LRPPRC protein was longer than 24 h in A2780 cells only but shortened to 8–12 h following exposure to 10 µM GAA (Additional file 5: Fig. S5C), indicating GAA decreased LRPPRC protein stability. The treatment of proteasome inhibitor MG132 or lysosomal inhibitors chloroquine (CQ) could not rescue the GAA-induced LRPPRC degradation, indicating this process was lysosomal and proteasome independent (Additional file 5: Fig. S5D, E). Previous studies reported lon peptidase 1 (Lonp1) that the interactions between SLIRP and the LRPPRC protein are degraded by mitochondrial matrix proteases, such as lon peptidase 1 (Lonp1), but little is known about the complex in OC cells. We observed no changes in SLIRP or Lonp1 in A2780 cells treated with GAA. Data from the co-IP assay showed that LRPPRC interacts with SLIRP, while GAA recruits Lonp1 for binding to LRPPRC, resulting in its degradation (Additional file 5: Fig. S5F).

At last, we performed a CCK8 assay in OC cells before and after the LRPPRC knockdown, in which the sensitivity of GAA was significantly reduced after the LRPPRC knockdown. Therefore, all these results demonstrated that GAA could directly bind to the OXPHOS controller LRPPRC and induced the antitumor effect of GAA was particle dependent on LRPPRC expression (Fig. 4G).

### GAA degrades LRPPRC and suppresses OXPHOS complex synthesis in OC cells in vitro and in vivo

**Fig. 5:**
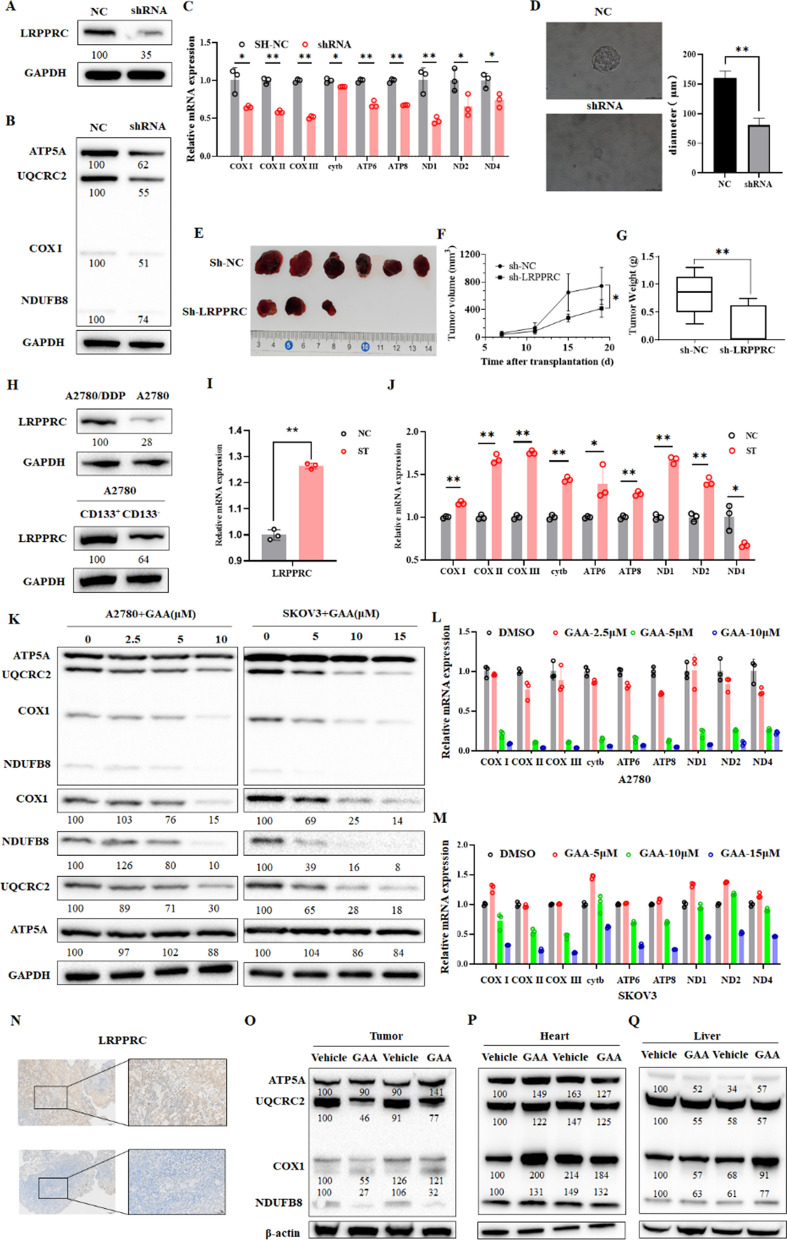
GAA suppresses LRPPRC and OXPHOS complex synthesis in OC cells in vitro and in vivo. **A** Silencing of LRPPRC via specific shRNA. **B** Western blot of the levels of OXPHOS-related proteins in A2780 cells with LRPPRC knockdown. **C** RT-PCR analysis of OXPHOS-related gene levels in A2780 cells with LRPPRC knockdown. **D** Cell spheroidization assay of A2780 cells with LRPPRC knockdown. **E**–**G** Image showing tumor inhibition by LRPPRC. Tumor weights and volumes were measured. **H** Western blot of LRPPRC levels in cisplatin (DDP)-resistant and cancer stem cells. **I** RT-PCR detection of LRPPRC levels in cancer stem cells. **J** RT-PCR detection of OXPHOS-related genes in cancer stem cells. **K** Western blot of OXPHOS-related proteins in A2780 and SKOV3 cells treated with GAA. **L**, **M** RT-PCR analysis of levels of OXPHOS-related genes in A2780 and SKOV3 cells treated with GAA. **N** IHC results of the staining patterns of LRPPRC. **O-Q** Western blot of protein levels of ATP5, NDUFB8, UQCRC2 and COX I in different regions of mice

LRPPRC participates in the OXPHOS mainly by increasing the expression of mtDNA but does not directly affect the electron transport chain activity of OXPHOS. Therefore, GAA-induced oxidative phosphorylation inhibition by targeting LRPPRC may be a novel mode of action that differs from traditional oxidative phosphorylation drugs. However, the roles of LRPPRC in regulating OXPHOS and tumor progression in OC remain unknown. LRPPRC knockdown A2780 cells (Fig. 5A) showed decreased mRNA levels of OXPHOS-related genes (Fig. 5C), including ND1, ND2 of complexes I, CYTB in complexes III, COX-I, COX-II, and COX-III in complexes IV, and ATP6 and ATP8 in complex V. Meanwhile, Immunoblotting results showed OXPHOS related proteins (ATP5A, UQCRC2, COX I and NDUFB8) were decreased after LRPPRC knockdown (Fig. 5B). In the suspension culture system, LRPPRC knockdown significantly decreased the number of tumor spheres, and the diameter of tumor spheroids was decreased by nearly 50% after LRPPRC knockdown (Fig. 5D). Transplantation of A2780 cells with stable shRNA-mediated knockdown of LRPPRC in our mouse model also led to remarkable inhibition of tumorigenicity, with an average suppression rate of 43.9% according to tumor volume. Moreover, 3 of the 6 mice did not develop tumors when transplanted with LRPPRC knockdown cells (Fig. 5E–G). Therefore, LRPPRC promoted the new mitochondrial OXPHOS synthesis, which is essential for tumor stem cell maintenance and in vivo tumorigenesis. Cancer stem cells play a key role in tumor development, drug resistance, and metastasis recurrence. These stem cells were reported to apply OXPHOS to maintain stemness. To determine whether LRPPRC is correlated with stemness and drug resistance in OC, we examined its expression in cancer stem cell (CSC)-like and cisplatin (DDP)-resistant cells. In CSC-like side populations as well as DDP-resistant A2780 cells, expression of LRPPRC was elevated (Fig. 5H, I). Moreover, OXPHOS-related genes were highly expressed in CSC-like cells (Fig. 5J). Therefore, LRPPRC promoted OXPHOS in OC and enhanced its ability to maintain CSC maintenance and form tumors in vivo.

Our PCR quantification also showed a significant decrease in mtDNA encoded OXPHOS complex subunits after GAA treatment, including ND1, ND2 in complex I; CYTB in complex III; COX-I, COX-II, and COX-III in complex IV; ATP6 and ATP8 in complex V (Fig. 5L, M). Western blotting further verified the decreased expression of mtDNA encoded proteins caused by GAA. GAA resulted in a significant decrease in the protein level of mtDNA-encoded COX-I. In addition, the protein levels of NDUFB8 and UQCRC2, which are encoded by nuclear genes, were also decreased (Fig. 5K). This is due to the dependence of their stability on the mtDNA-encoded OXPHOS complex subunits. Previous studies have also demonstrated that GAA could act as an inhibitor of the antiapoptotic protein Bcl-2. A2780 cells treated with ABT737 (a Bcl-2 inhibitor) showed no significant decline in OXPHOS-related mRNA (Additional file 5: Fig. S4). Therefore, GAA could induce OXPHOS complex synthesis defect in OC cells, and this effect was LRPPRC-dependent.

We also tested the expression of the LRPPRC protein and OXPHOS complex in vivo before and after GAA treatment. Tumors received GAA management showed a downregulated expression of LRPPRC and OXPHOS complex subunits (Fig. 5N, O). Whereas the protein level of OXPHOS complex subunits remained unchanged in normal tissues, including the heart and liver (Fig. 5P, Q). Therefore, in vivo, experiments demonstrated that targeting LRPPRC protein by GAA can achieve tumor cell-specific oxidative phosphorylation inhibition.

### LRPPRC is overexpressed in OC and represents a new prognostic marker and therapeutic target

**Fig. 6:**
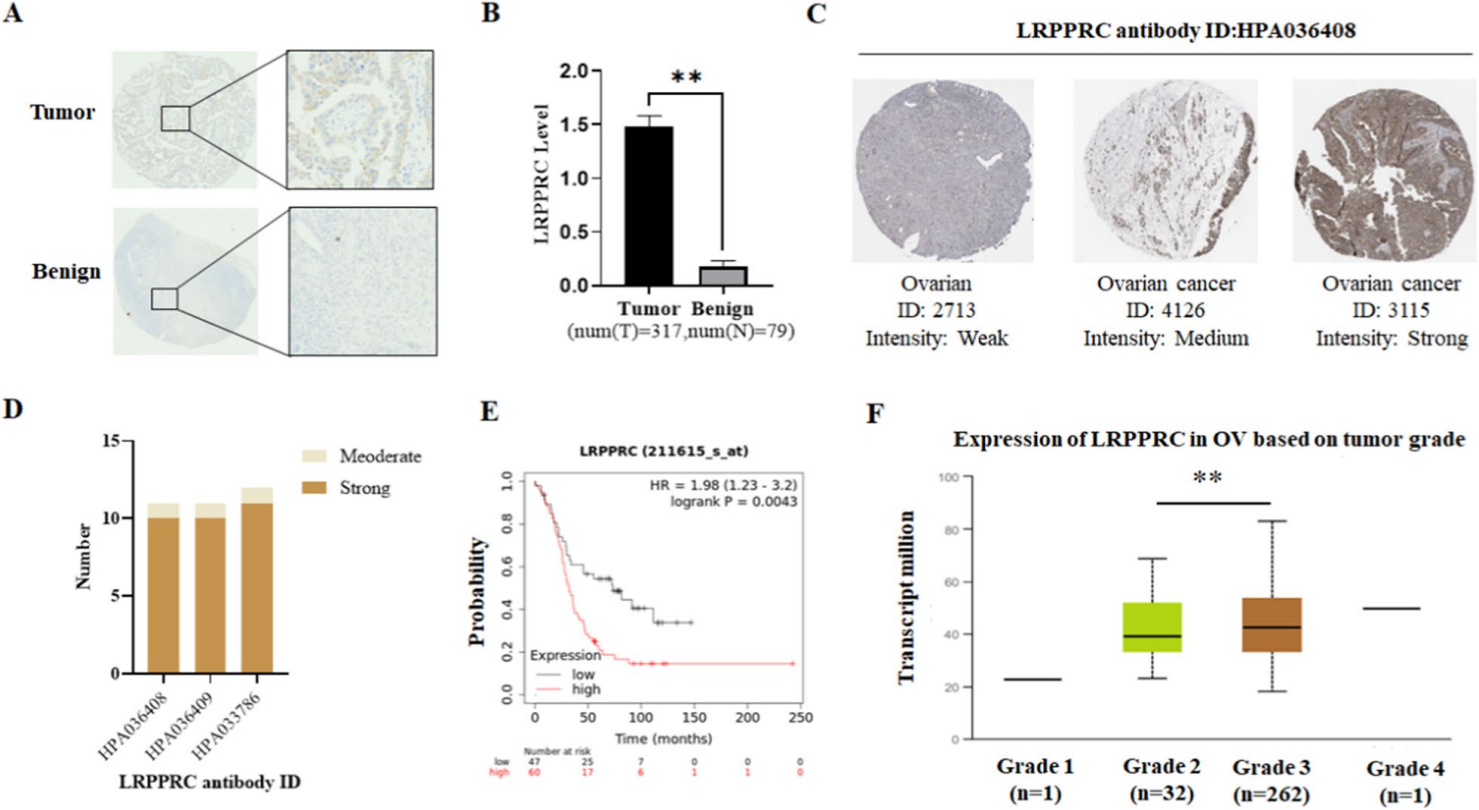
LRPPRC is overexpressed in OC and represents a new prognostic marker and therapeutic target. **A**, **B** IHC detection of LRPPRC protein levels in advanced serous OC (n = 317) and benign ovarian cysts (n = 79). **C**, **D** Human protein atlas data showed that LRPPRC was overexpressed in ovarian cancer **E** Kaplan-Meier survival analysis of association of LRPPRC expression with overall survival of patients in the TCGA ovarian cancer dataset. **F** LRPPRC levels were correlated with tumor stage

To determine the correlations between LRPPRC and ovarian cancer, we compared its protein expression level in advanced serous OC ( n = 317) and benign ovarian cysts (n = 79) by IHC. LRPPRC expression was markedly increased in cancer tissue samples relative to benign tissues. More specifically, 61% of tumor tissues (192 of 317) were LRPPRC positive, which was significantly higher than that in benign ovarian cysts (14%, 11 of 79) (Fig. 6A, B). And the human protein atlas data showed a similar result, in which a strong positive signal was found in most of the ovarian cancer tissues but not in normal ovarian tissue (Fig. 6C, D). We additionally analyzed the correlation between LRPPRC expression and overall survival (OS) of 107 patients with ovarian cancer from previously generated microarray data sets. High LRPPRC expression was associated with poor clinical outcomes (HR = 1.98, *P* = 0.0043) (Fig. 6E) and tumor stage (Fig. 6F) in patients with OC. The collective findings support the role of the LRPPRC in promoting OC progression.

## Discussion

Earlier reports suggest that a decrease in respiration and ATP production under conditions of mitochondrial function injury is mainly caused by the Warburg effect [22, 23]. This view has been increasingly questioned by growing evidence that several tumors are highly dependent on OXPHOS for survival [24, 25]. In our study, the traditional therapeutic drug, GAA, screened from a natural compound library, inhibited mitochondrial respiration in OC cells and targeted LRPPRC to affect energy metabolism. LRPPRC was highly expressed in OC tissue and negatively correlated with patient prognosis. Knockdown of LRPPRC expression resulted in significant inhibition of OC cell proliferation. To our knowledge, this is the first study to document the involvement and mechanism of action of LRPPRC in the progression of ovarian cancer.

Resistance to therapy and metastasis are major clinical challenges, and ovarian cancer development is accompanied by distinct changes in cellular metabolism, such as significant upregulation of OXPHOS [26–28]. To ascertain the effects of drugs on OXPHOS levels in ovarian cancer, we used the Mito tracker to detect the mitochondrial membrane potential in OC cells. GAA exerted the most significant inhibitory effects on OC cells. Consistently, proteomic results confirmed that GAA predominantly affects OXPHOS.

While previous studies have shown that GAA affects the stability of the LRPPRC protein in lung cancer cells [29], its therapeutic value in ovarian cancer is yet to be ascertained. Mutations in LRPPRC can lead to Leigh syndrome, French-Canadian type (LSFC), a human disorder characterized by neurodegeneration and cytochrome c oxidase deficiency [30–32]. High expression of LRPPRC is related to poor prognosis in prostate cancer patients, consistent with data obtained with gastric cancer [33–35]. In this context, our experiments showed that LRPPRC expression is increased in ovarian cancer tissues and negatively correlated with OS. Interestingly, LRPPRC was highly expressed in drug-resistant OC and CD133^+^ OC cells. Knockdown of LRPPRC led to decreased levels of OXPHOS and tumorigenicity in OC cells, supporting the utility of LRPPRC suppression as a novel strategy for ovarian cancer management.

GAA, a traditionally used natural drug product, inhibits the hormone receptor, hormone synthase, and tumor cell growth and regulates ovarian hormones [36, 37]. In previous studies by our group, GAA was shown to act as a specific LRPPRC knockdown agent, both in vitro and in vivo. However, its mechanism of action in ovarian cancer is currently unknown. Consistent with these findings, experiments from the current study demonstrated that GAA affects the stability of the LRPPRC protein, not via ubiquitination or autophagy but through modulating interactions of LRPPRC with LONP1 via SLIRP. In animal experiments, GAA exerted significant growth inhibitory effects against ovarian cancer with reduced side effects. Additionally, GAA suppressed OXPHOS-related protein levels in tumors but had no effects on the liver or heart. Our results suggest that GAA acts specifically on tumor tissue, which could explain its low toxicity and side effects.

**Fig. 7:**
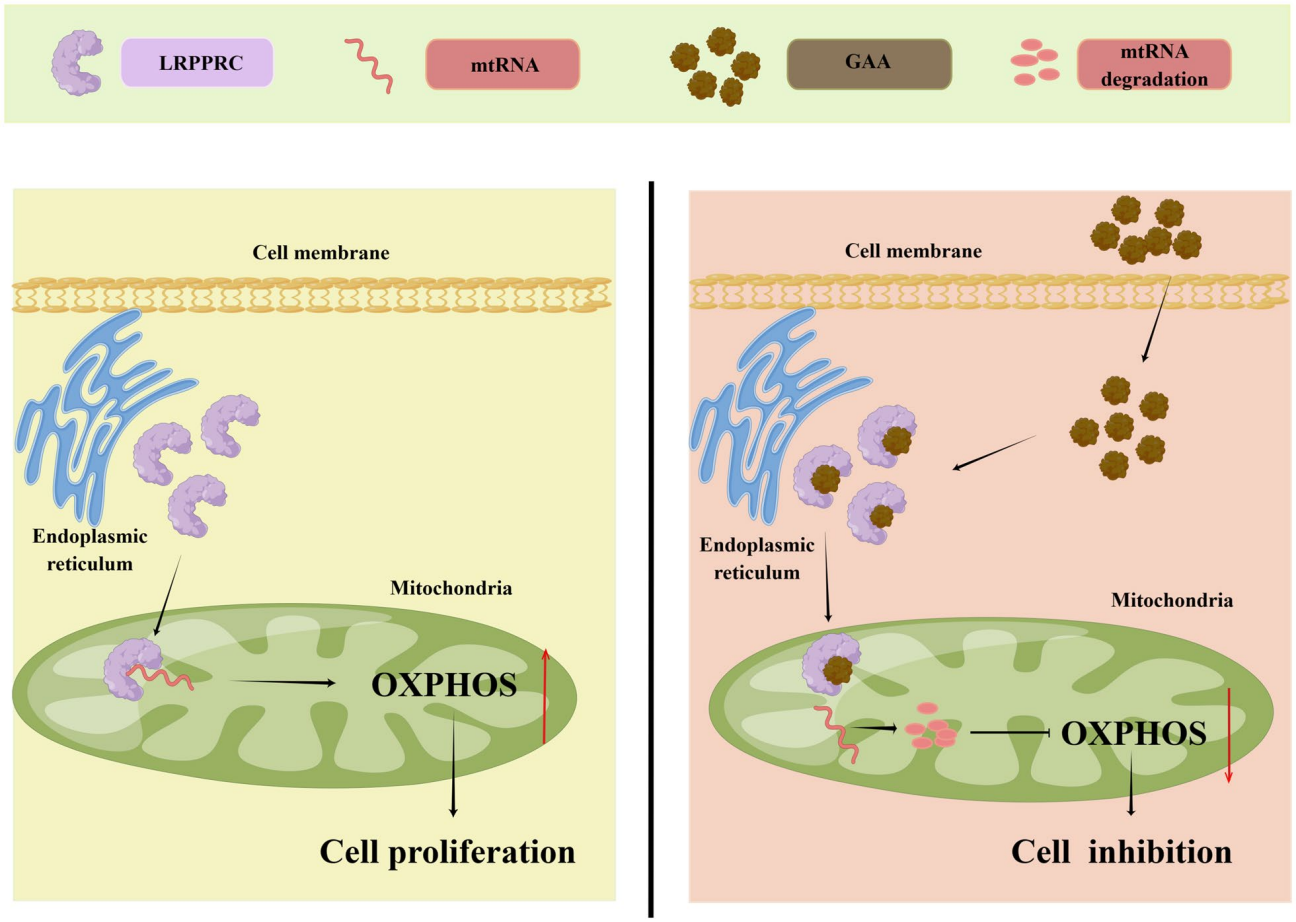
GAA inhibits cell proliferation through LRPPRC regulated OXPHOS. LRPPRC interacts with mtRNA, and this interaction increases mtRNA’s stability, thereby promoting the synthesis of oxidative phosphorylation complex subunits during cancer cell proliferation. Competitive binding of GAA and mtRNA to LRPPRC destabilized both mtRNA and LRPPRC simultaneously. This inhibition suppressed the synthesis of oxidative phosphorylation complex subunits, resulting in OXPHOS defects, growth inhibition, and stem cell clearance

 In conclusion, we have identified a protein biomarker and its inhibitor for targeted therapy of ovarian cancer for the first time. GAA inhibits cell proliferation and affects OXPHOS in OC cells through the degradation of the LRPPRC protein, supporting its potential utility as a natural therapeutic agent for ovarian cancer (Fig. 7). The therapeutic value of LRPPRC should also be considered. Overall, the current study provides valuable insights into the mechanism of action of OXPHOS in ovarian cancer mediated by LRPPRC and highlights novel strategies for ovarian cancer therapy.

## Supplementary information

**Additional file 1: Table S1.** Candidate natural compounds.

**Additional file 2. Data 1.** Inhibition rate of natural compounds on ovarian cancer cells.

**Additional file 3. Data 2**. Primer sequence.

**Additional file 4.** Experimental procedure.

**Additional file 5: Fig. S1.** Mito tracker staining after A2780 cell was treated with candidate natural compounds. **Figure S2.** GAA suppresses the malignant phenotypes of OC cells in vitro. (A) Wound healing assay of SKOV3 cells treated with GAA for 0 h, 24 h and 48 h. (B) Cell spheroidization assay of SKOV3 cells treated with GAA. (C) Clonogenic assays of SKOV3 cells treated with GAA for 48 h. (D) Flow cytometry analysis showing cell cycle changes in SKOV3 cells treated with GAA for 48 h. **Figure S3.** GAA suppresses the proliferation of OC but not apoptotic. (AB) Flow cytometry analysis showing cell apoptosis changes in OC cells treated with GAA for 48 h. **Figure S4.** RT-PCR detected the levels of OXPHOS related genes after A2780 cell treated with Bcl-2 inhibitor (ABF737). **Figure S5.** GAA induce LRPPRC degradation in OC cells. (A-B) RT-PCR detected the mRNA levels of LRPPRC after cells treated with GAA. (C) Immunoblotting of LRPPRC in A2780 cell treated with GAA (10 μM) and CHX (2.5 μg/ml) in different time point. (D) Immunoblotting of LRPPRC in A2780 cell treated with GAA (10 μM) and CQ (20 μM) in different time point. (E) Immunoblotting of LRPPRC in A2780 cell treated with GAA (10 μM) and MG132 (10 μM) in different time point. (F) Enrichment of Lonp1 and SLIRP in immunoprecipitation (IP) using LRPPRC special antibody followed by Western-blot in the presence of different concentrations of GAA with A2780 cell.

## Data Availability

All data generated or analyzed during this study are included in the manuscript and its additional information files.

## Abbreviations

OC: Ovarian cancer
OXPHOS: Oxidative phosphorylation
GAA: Gossypol acetic acid
LRPPRC: Leucine-rich pentatricopeptide repeat containing protein
PDC: Patient-derived cell
LC-MS: Liquid Chromatograph Mass Spectrometer
IHC: Immunohistochemistry
TCA: Tricarboxylic acid cycle
WB: Western-blot
BUN: Blood urea nitrogen
AST: Aspartate aminotransferase
CRE: Creatinine
ALT: Alanine aminotransferase
OCR: Oxygen consumption rate
ECAR: Extracellular acidification rate
