# Supplementary figures and images for: A traditional gynecological medicine inhibits ovarian cancer progression and eliminates cancer stem cells via the LRPPRC–OXPHOS axis
Source: J Transl Med. 2023 Jul 26;21:504. doi: 10.1186/s12967-023-04349-3 (PMC10373366; doi:10.1186/s12967-023-04349-3)

**Table S1：Candidate natural compounds**


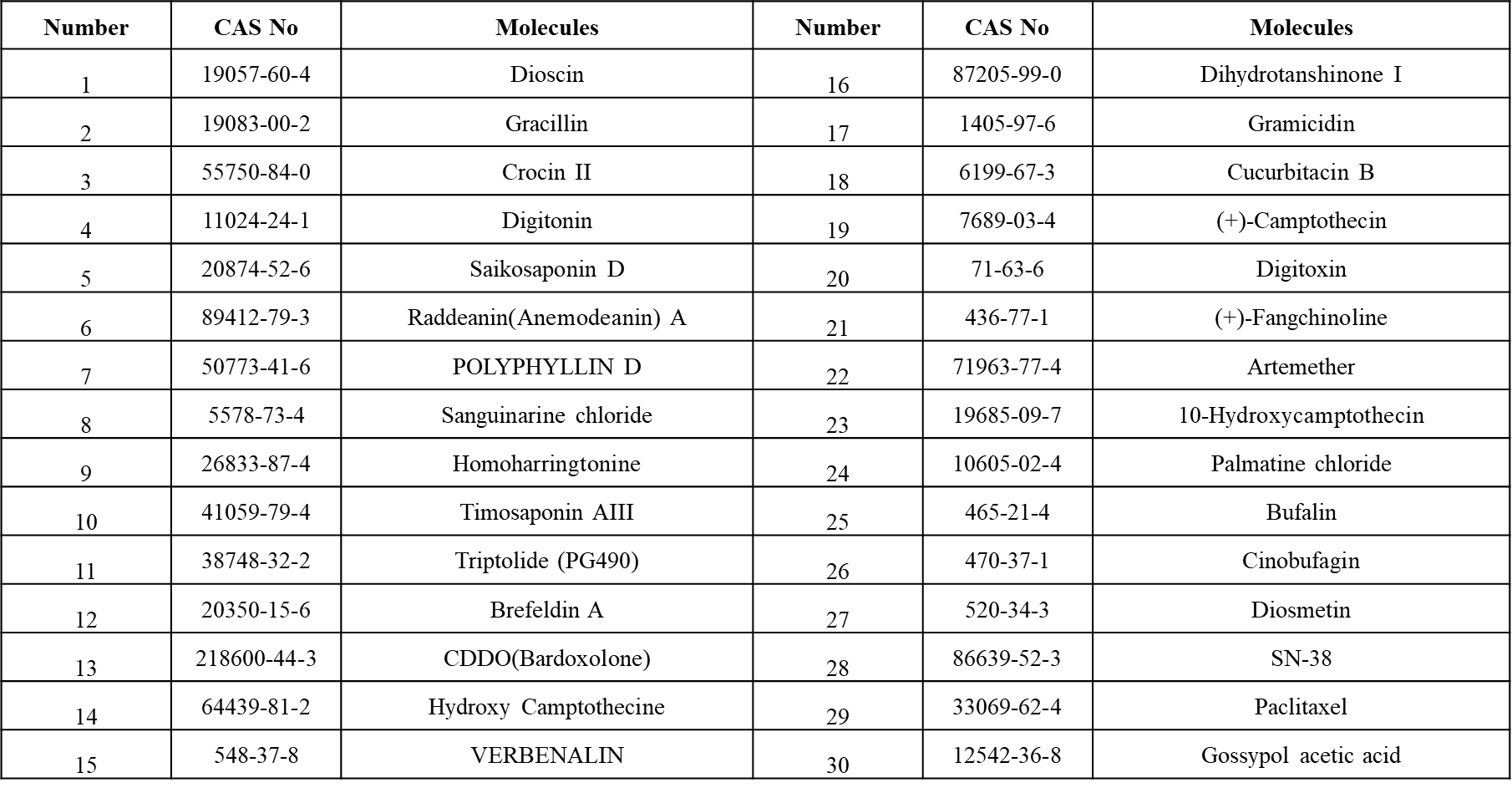

Supplement: Supplementary file 1 — Additional file 1: Table S1. Candidate natural compounds. [file 12967_2023_4349_MOESM1_ESM.docx]
