# Supplementary material for: A traditional gynecological medicine inhibits ovarian cancer progression and eliminates cancer stem cells via the LRPPRC–OXPHOS axis
Source: J Transl Med. 2023 Jul 26;21:504. doi: 10.1186/s12967-023-04349-3 (PMC10373366; doi:10.1186/s12967-023-04349-3)
Supplement: Supplementary file 4 — Additional file 4. Experimental procedure. [file 12967_2023_4349_MOESM4_ESM.docx]

**Metabolite extraction**

Take out the sample at -80 ℃, add 500 ml of methanol acetonitrile aqueous solution(2:2:1, v/v)respectively, vortex for 60 sec, low temperature ultrasound for 30 min for twice time, place at -20 ℃ for 1hour to precipitate protein, 14000rpm, centrifuge at 4 ℃ for 20min, take the supernatant and freeze dry.

**LC-MS analysis**

*HPLC condition*

The sample was separated by Agilent 1290 Infinity LC ultra-high performance liquid chromatography system. Mobile phase: liquid A is 10 mM ammonium acetate aqueous solution, liquid B is acetonitrile. The sample is placed in a 4 ℃ automatic sampler, the column temperature is 45 ℃, and the flow rate is 300 μL/min, injection volume 2 μL. The relevant liquid phase gradient is as follows: 0-18 min, liquid B changes linearly from 90% to 40%; 18-18.1 min, liquid B changed linearly from 40% to 90%; 18.1-23 min, liquid B maintained at 90%. In the sample queue, one QC sample is set for each certain number of experimental samples at an interval to detect and evaluate the stability and repeatability of the system; The standard mixture of energy metabolism substances is set in the sample queue for correction of chromatographic retention time.

*Mass spectrometric analysis*

The 5500 QTRAP mass spectrometer (AB SCIEX) was used for mass spectrometry analysis in negative ion mode. 5500 QTRAP ESI source conditions are as follows: source temperature 450 ℃, ion Source Gas1 (Gas1): 45, Ion Source Gas2 (Gas2): 45, Current gas (CUR): 30, ionSapry Voltage Floating (ISVF) - 4500 V; Use MRM mode to detect ion pairs to be measured.

**Data analysis**

Multiquant software was used to extract the chromatographic peak area and retention time. The retention time was corrected with the standard of energy metabolism substances, and the metabolites were identified.
