# Supplementary material for: A traditional gynecological medicine inhibits ovarian cancer progression and eliminates cancer stem cells via the LRPPRC–OXPHOS axis
Source: J Transl Med. 2023 Jul 26;21:504. doi: 10.1186/s12967-023-04349-3 (PMC10373366; doi:10.1186/s12967-023-04349-3)
Supplement: Supplementary file 5 — Additional file 5: Fig. S1. Mito tracker staining after A2780 cell was treated with candidate natural compounds. Figure S2. GAA suppresses the malignant phenotypes of OC cells in vitro. (A) Wound healing assay of SKOV3 cells treated with GAA for 0 h, 24 h and 48 h. (B) Cell spheroidization assay of SKOV3 cells treated with GAA. (C) Clonogenic assays of SKOV3 cells treated with GAA for 48 h. (D) Flow cytometry analysis showing cell cycle changes in SKOV3 cells treated with GAA for 48 h. Figure S3. GAA suppresses the proliferation of OC but not apoptotic. (AB) Flow cytometry analysis showing cell apoptosis changes in OC cells treated with GAA for 48 h. Figure S4. RT-PCR detected the levels of OXPHOS related genes after A2780 cell treated with Bcl-2 inhibitor (ABF737). Figure S5. GAA induce LRPPRC degradation in OC cells. (A-B) RT-PCR detected the mRNA levels of LRPPRC after cells treated with GAA. (C) Immunoblotting of LRPPRC in A2780 cell treated with GAA (10 μM) and CHX (2.5 μg/ml) in different time point. (D) Immunoblotting of LRPPRC in A2780 cell treated with GAA (10 μM) and CQ (20 μM) in different time point. (E) Immunoblotting of LRPPRC in A2780 cell treated with GAA (10 μM) and MG132 (10 μM) in different time point. (F) Enrichment of Lonp1 and SLIRP in immunoprecipitation (IP) using LRPPRC special antibody followed by Western-blot in the presence of different concentrations of GAA with A2780 cell. [file 12967_2023_4349_MOESM5_ESM.docx]

**Additional information**

**A traditional gynecological medicine inhibits ovarian cancer progression and eliminates cancer stem cells via the LRPPRC–OXPHOS axis**

Ruibin Jiang^1^, Zhongjian Chen^1^, Maowei Ni^1^, Xia Li^1^, Hangjie Ying^1^, Jianguo Fen^1^, Danying Wan^1^, Chanjuan Peng^1^, Wei Zhou^2, #^, Linhui Gu^1, #^.

^1^ Zhejiang Cancer Hospital, Hangzhou Institute of Medicine (HIM), Chinese Academy of Sciences, Hangzhou, Zhejiang 310022, China.

^2^ Hangzhou Institute of Medicine (HIM), Chinese Academy of Sciences, Hangzhou, Zhejiang 310022, PR China.

^#^Corresponding author: Linhui Gu, Email: [gulh@zjcc.org.cn](mailto:gulh@zjcc.org.cn)

Wei Zhou, Email: zhouwei1989@iccas.ac.cn


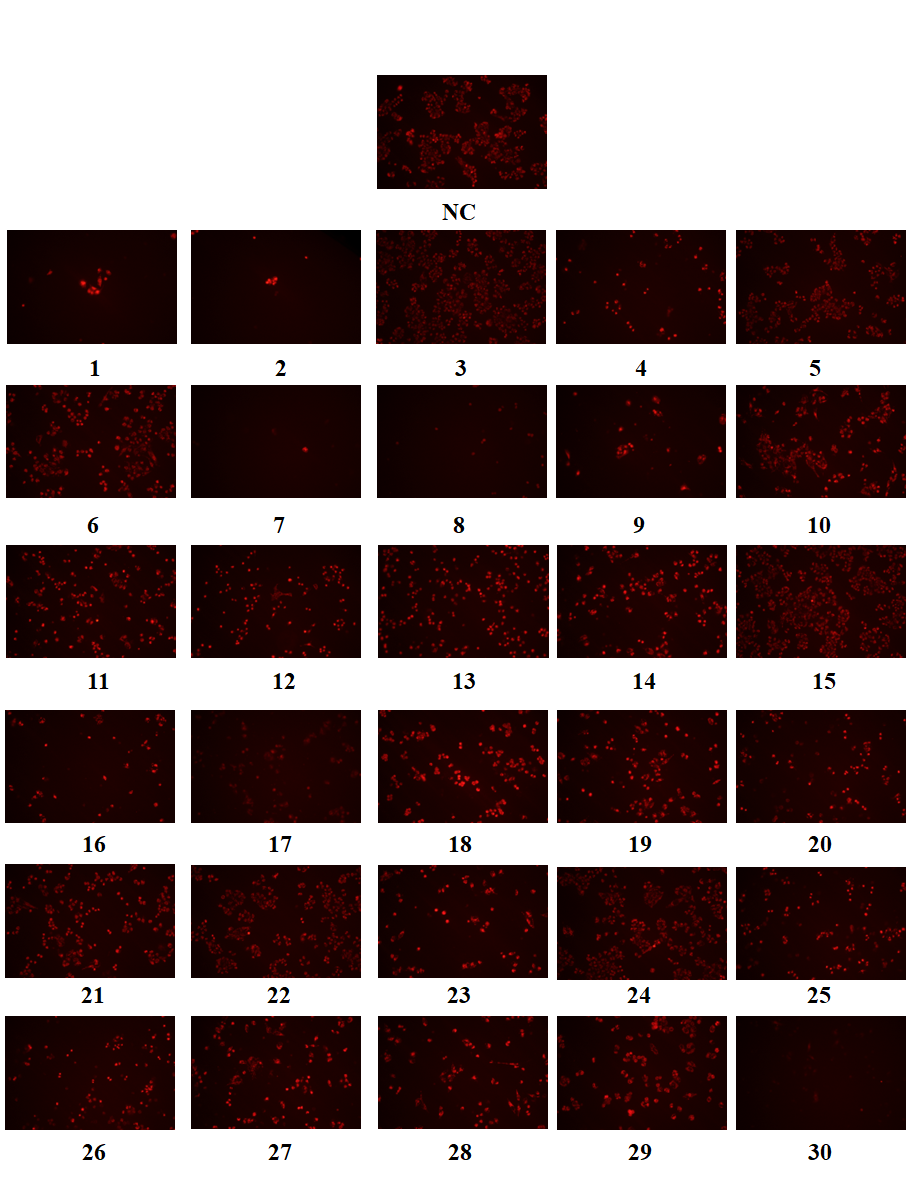


**Figure S1** Mito tracker staining after A2780 cell was treated with candidate natural compounds.


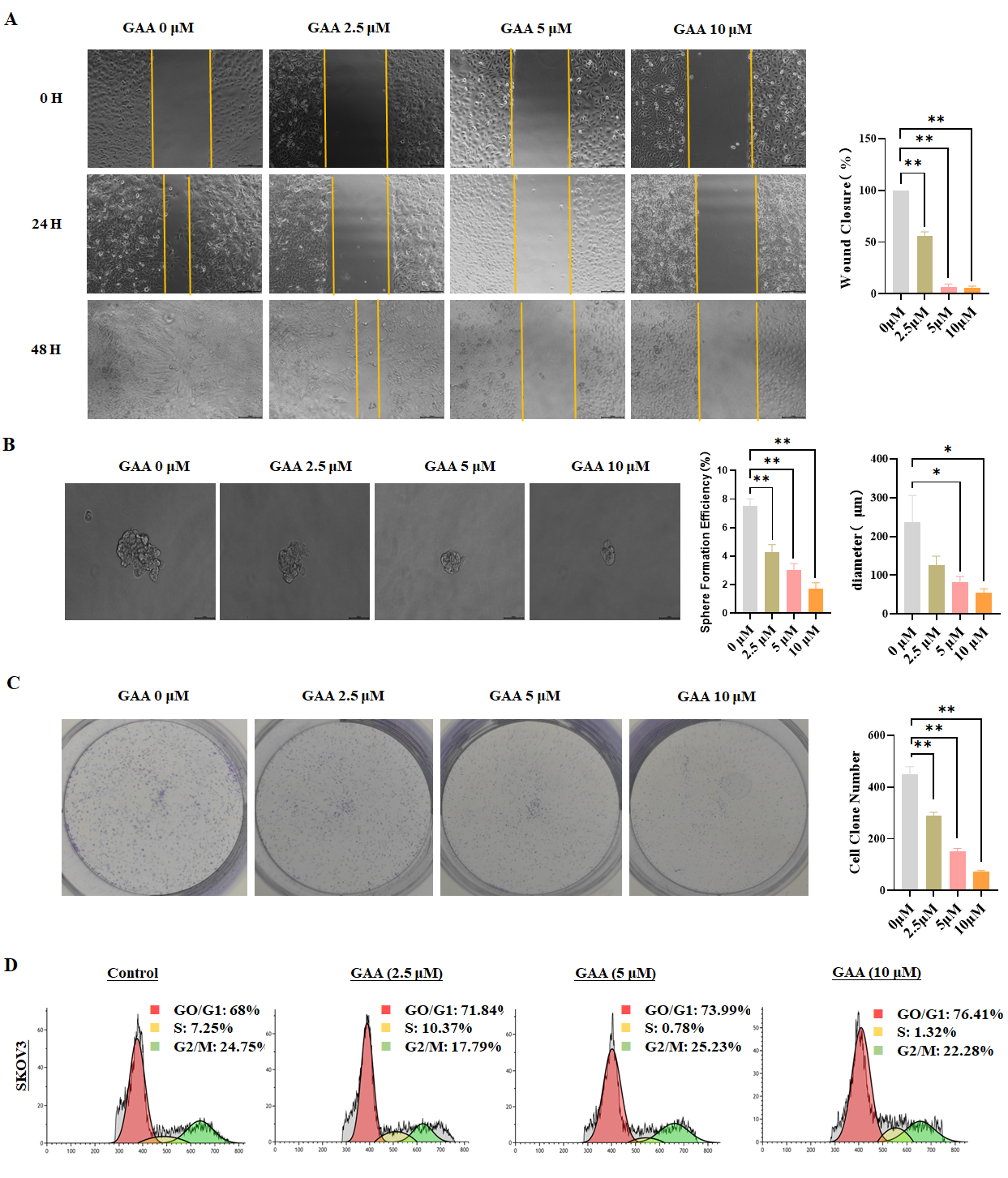


**Figure S2** GAA suppresses the malignant phenotypes of OC cells *in vitro*. (A) Wound healing assay of SKOV3 cells treated with GAA for 0 h, 24 h and 48 h. (B) Cell spheroidization assay of SKOV3 cells treated with GAA. (C) Clonogenic assays of SKOV3 cells treated with GAA for 48 h. (D) Flow cytometry analysis showing cell cycle changes in SKOV3 cells treated with GAA for 48 h.


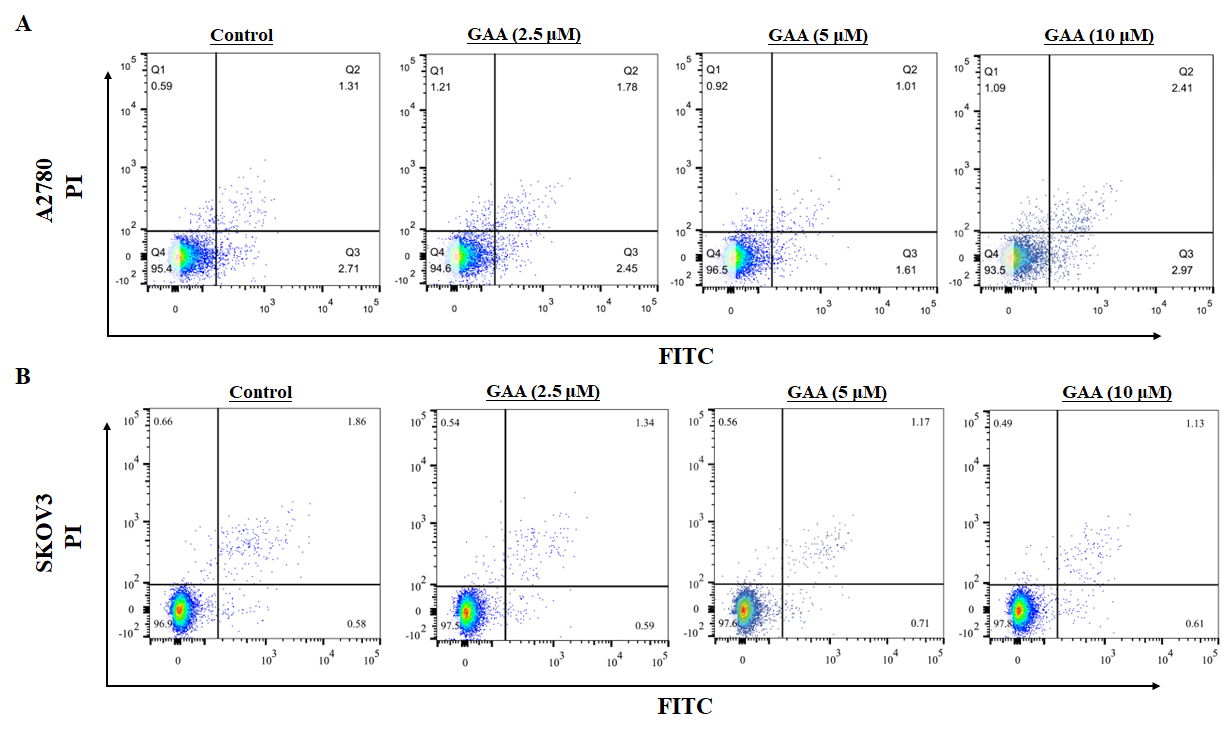


**Figure S3** GAA suppresses the proliferation of OC but not apoptotic. (AB) Flow cytometry analysis showing cell apoptosis changes in OC cells treated with GAA for 48 h.


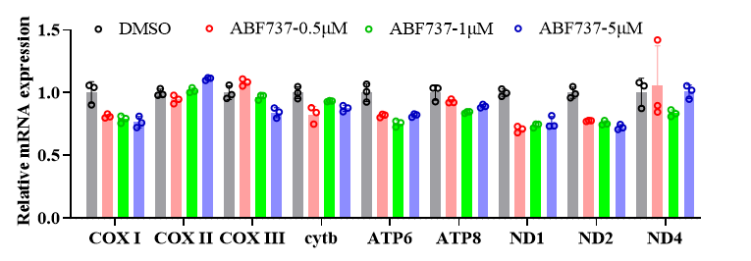


**Figure S4** RT-PCR detected the levels of OXPHOS related genes after A2780 cell treated with Bcl-2 inhibitor (ABF737).


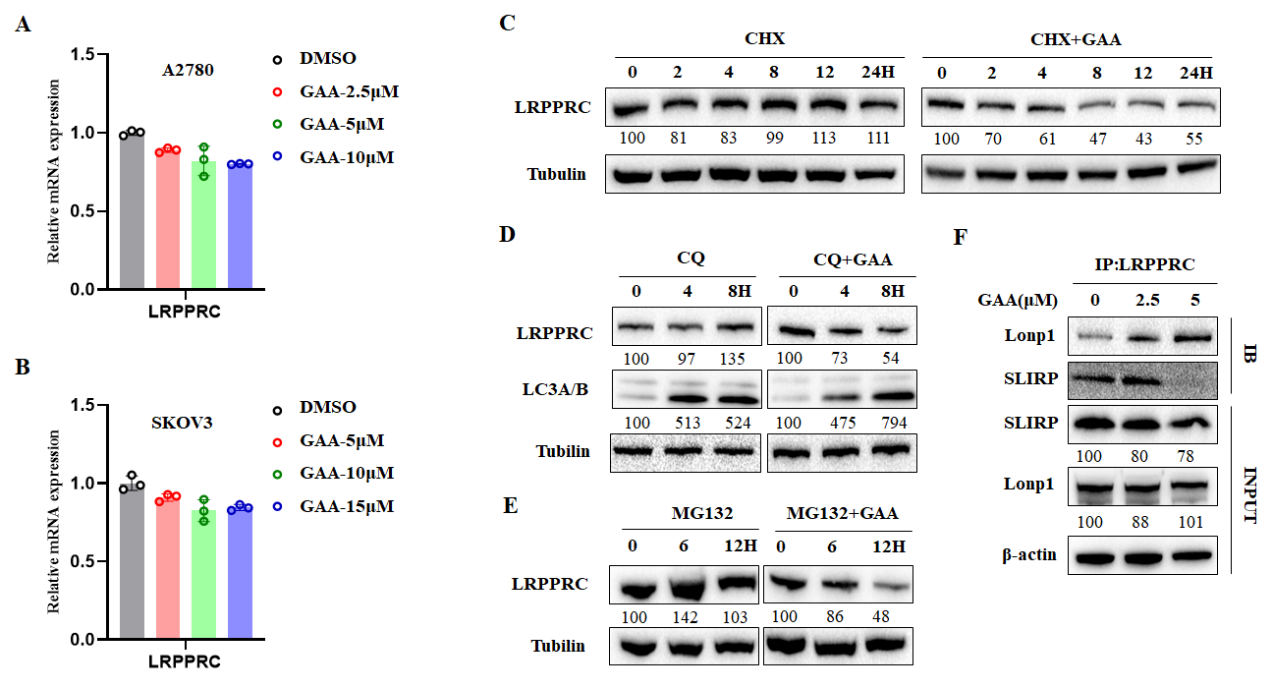


**Figure S5** GAA induce LRPPRC degradation in OC cells. (A-B) RT-PCR detected the mRNA levels of LRPPRC after cells treated with GAA. (C) Immunoblotting of LRPPRC in A2780 cell treated with GAA (10 μM) and CHX (2.5 μg/ml) in different time point. (D) Immunoblotting of LRPPRC in A2780 cell treated with GAA (10 μM) and CQ (20 μM) in different time point. (E) Immunoblotting of LRPPRC in A2780 cell treated with GAA (10 μM) and MG132 (10 μM) in different time point. (F) Enrichment of Lonp1 and SLIRP in immunoprecipitation (IP) using LRPPRC special antibody followed by Western-blot in the presence of different concentrations of GAA with A2780 cell.
